# The effects of naloxone, diazepam, and quercetin on seizure and sedation in acute on chronic tramadol administration: an experimental study

**DOI:** 10.1186/s12993-021-00178-w

**Published:** 2021-05-29

**Authors:** Samaneh Nakhaee, Khadijeh Farrokhfall, Ebrahim Miri-Moghaddam, Mohsen Foadoddini, Masoumeh Askari, Alireza Amirabadizadeh, Jeffrey Brent, Bruno Megarbane, Omid Mehrpour

**Affiliations:** 1 Medical Toxicology and Drug Abuse Research Center (MTDRC), Birjand University of Medical Sciences (BUMS), Birjand, Iran; 2 Cardiovascular Diseases Research Center, Birjand University of Medical Sciences, Birjand, Iran; 3 School of Medicine, University of Colorado, Aurora, CO USA; 4 Department of Medical and Toxicological Critical Care, Lariboisière Hospital; INSERM UMRS-1144; University of Paris, Paris, France; 5 Mel and Enid Zuckerman College of Public Health, University of Arizona, Tucson, AZ USA

**Keywords:** Quercetin, Diazepam, Naloxone, Neurotoxicity, Tramadol

## Abstract

**Background:**

Tramadol is a widely used synthetic opioid. Substantial research has previously focused on the neurological effects of this drug, while the efficacy of various treatments to reduce the associated side effects has not been well studied. This study aimed to evaluate the protective effects of naloxone, diazepam, and quercetin on tramadol overdose-induced seizure and sedation level in male rats.

**Methods:**

The project was performed with 72 male Wistar rats with an average weight of 200–250 g. The rats were randomly assigned to eight groups. Tramadol was administered intraperitoneally at an initial dose of 25 mg/kg/day. On the 14th day, tramadol was injected at 75 mg/kg, either alone or together with naloxone, diazepam, and quercetin (acute and chronic) individually or in combination. The rats were monitored for 6 h on the last day, and the number, the duration, and the severity of seizures (using the criteria of Racine) were measured over a 6-h observation period. The sedation level was also assessed based on a 4-point criterion, ranging from 0 to 3. Data were analyzed in SPSS software using Kruskal–Wallis, Chi-square, regression analysis, and generalized estimating equation (GEE) tests. The significance level was set at P < 0.05.

**Results:**

The naloxone-diazepam combination reduced the number, severity, and cumulative duration of seizures compared to tramadol use alone and reduced the number of higher-intensity seizures (level 3, 4) to a greater extent than other treatments. Naloxone alone reduced the number and duration of seizures but increased the number of mild seizures (level 2). Diazepam decreased the severity and duration of seizures. However, it increased the number of mild seizures (level 2). In comparison with the tramadol alone group, the acute quercetin group exhibited higher numbers of mild (level 2) and moderate (level 3) seizures. Chronic quercetin administration significantly increased the number of mild seizures. In the GEE model, all groups had higher sedation levels than the saline only group (P < 0.001). None of the protocols had a significant effect on sedation levels compared to the tramadol group.

**Conclusion:**

The combined administration of naloxone and diazepam in acute-on-chronic tramadol poisoning can effectively reduce most seizure variables compared to tramadol use alone. However, none of the treatments improved sedation levels.

## Background

Tramadol is widely used worldwide as a centrally-acting analgesic to treat moderate to severe pain [1]. Given the increasing numbers of tramadol overdose and fatalities in recent decades, this drug has been classified as a controlled substance in several countries [2]. Tramadol overdose can lead to loss of consciousness, seizures, respiratory depression, serotonin syndrome, and death [3, 4]. However, the molecular and biochemical mechanisms of tramadol toxicity are still poorly understood [5]. Recent studies have shown that tramadol increases oxidative stress in various body tissues, including the brain; however, the extent to which this occurs in acute toxicity, if any, is unknown [6]. Nevertheless, tramadol overdose has been shown to cause brain congestion, edema, and inflammatory infiltrates in the rat [7]. Chronic tramadol administration at a dose of 50 mg/kg causes histological abnormalities demonstrating oxidative stress-related apoptosis in the rat cortex [8, 9]. Similarly, chronic low but escalating tramadol doses have also been associated with neuronal degeneration in the rat model [10].

Tramadol causes self-limiting tonic–clonic seizures within 4 to 6 h after administration, although electroencephalographic (EEG) changes, recurrent seizures or persistent epilepsy are not uncommon [11]. Tramadol-induced seizures appear to be dose-independent and may occur even within the recommended treatment range, although they are clearly more common when patients exceed recommended doses [12]. The cause of tramadol-induced seizures is not well understood. Nevertheless, based on animal study findings, seizures appear to be unlikely to be related to the serotonergic effects of tramadol [13]. Alternative pathways involving opioid, histamine, glutaminergic, or gamma-aminobutyric acid (GABA) receptors have been implicated in tramadol seizurgenesis [4]. It is well established that inhibition of GABAergic neurons or activation of glutamatergic neurons leads to seizures. Tramadol, and its metabolite O-desmethyl tramadol (M1), inhibits (GABA)-A receptors at high concentrations and glutaminergic *N*-methyl-d-aspartate (NMDA) receptors at clinically relevant concentrations [14].

Naloxone is an opioid receptor antagonist that reverses opioid-induced respiratory depression. However, there are conflicting findings on its use in tramadol poisoning and its effect on seizures [12]. Previous experimental and human investigations have shown that naloxone may reduce [12, 15], increase [4, 16, 17], or have no impact [18] on the risk of tramadol-induced seizures. Benzodiazepines act as a GABA-A/B receptor enhancer and, thus, may tend to protect against and reverse seizures due to tramadol [3, 14]. Experimental studies have demonstrated that benzodiazepines are effective in the prophylaxis of tramadol overdose-induced seizures [3], and more limited seizure activity has been reported in patients co-ingesting tramadol and benzodiazepines [17]. In contrast, fatalities have been reported with the co-ingestion of benzodiazepines and tramadol [19], although the exact mechanisms of such drug-drug interactions are not fully known.

Found in vegetables and fruits, quercetin is a flavonoid with many attributed therapeutic and protective properties, including antioxidant and anti-inflammatory activities [20, 21]. Neuroprotective effects of quercetin on various central nervous system disorders such as memory impairment [22, 23] and seizures [20, 21, 24] have been reported. An animal study investigating the effect of quercetin on GABA-a5 receptor gene expression in kainic acid-induced seizures indicated increased expression of this gene in the hippocampus of the kainic acid group, whereas expression decreased in the quercetin group [20]. Protective effects of acute [24] and chronic [20] quercetin administration against drug-induced seizures has been reported in some studies. However, the protective effects of acute and chronic dosing in the setting of tramadol overdose have remained understudied.

To our knowledge, no study investigating the acute-on-chronic neurological effects of tramadol is available. This is important because patients who use tramadol chronically may intentionally or accidentally overdose. Moreover, the potential protective effects of quercetin, diazepam, and naloxone on the nervous system in acute-on-chronic use of tramadol are unknown. This study aimed to investigate the protective effects, if any, of quercetin, diazepam, and naloxone on tramadol-induced toxicity in an acute-on-chronic male rat model.

## Methods

This study was performed with 72 male Wistar rats (body weight, 200–250 g, age, 12 weeks) purchased from the department for keeping laboratory animals at Birjand University of medical sciences (BUMS). All experiments were performed as per the international rules for laboratory animals and in conditions agreed by the Research Centre of Experimental Medicine, BUMS. Rats were kept at constant room temperature [22 ± 2 °C, light/dark 12 h intervals] and free access to food and water in the BUMS animal facility. The BUMS ethics committee approved the study protocol (identifier: IR.BUMS.REC.1397.194). Healthy Wistar rats with normal behavior and activity were included. Previously used rats from other experimental studies were not included. Exclusion criteria were considered as death during the experiment.

Quercetin, naloxone, diazepam, and pentobarbital were purchased from the Sigma-Aldrich Company, and tramadol was obtained from the Temad Company (Iran, Karaj). Quercetin, diazepam, and naloxone were dissolved in DMSO, and tramadol in normal saline. The concentration of DMSO in this study was 2%. The injection volume was 1 ml/kg. The DMSO was administrated in the inert dose range for behavioral [25] and experimental animal studies [26, 27]. As previously demonstrated, tramadol doses were selected to allow the onset of typical tramadol overdose features in the rats [4]. Other drugs were given at their usual pharmacologic doses based on previous literature [4]. The dose of quercetin was that which is effective in reducing seizures as previously reported [20].

**Fig. 1:**
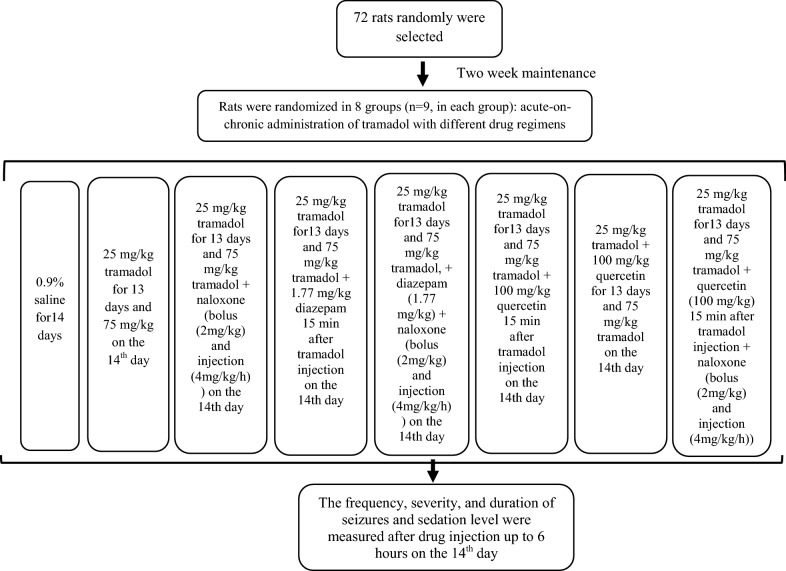
Flow diagram of different experiments and the study process

The animals were randomly assigned to 8 groups of 9 animals each after two week of adaptation to laboratory conditions. The calculation of sample size is based on the “resource equation approach” design for animal studies using the formula of N = (10/*k* + 1) × *k*, where *N* = total number of subjects, *k* = number of groups [28]. The first group (*Control*) received 0.9% saline intraperitoneally for 14 days. The second group (*Tramadol*) received 25 mg/kg tramadol IP for 13 days and 75 mg/kg tramadol IP on the 14th day. Group 3 (*Naloxone*) received 25 mg/kg tramadol IP for 13 days and 75 mg/kg tramadol IP on the 14th day, along with a 2 mg/kg naloxone intravenously (IV) bolus 15 min after tramadol injection, followed by 4 mg/kg naloxone injection once every hour for 6 h [4]. Group 4 (*Diazepam*) received 25 mg/kg tramadol IP for 13 days and 75 mg/kg tramadol IP on the 14th day along with 1.77 mg/kg of diazepam IP 15 min after tramadol injection. Group 5 (*Naloxone-Diazepam*) received 25 mg/kg tramadol IP for 13 days and 75 mg/kg tramadol IP on the 14th day, together with 1.77 mg/kg diazepam IP and 2 mg/kg naloxone IV followed by 4 mg/kg naloxone injection once every hour for 6 h. Group 6 (*Acute Quercetin*) received 25 mg/kg tramadol IP for 13 days and 75 mg/kg tramadol IP on the 14th day, followed by 100 mg/kg quercetin IP, 15 min after tramadol injection. Group 7 (*Chronic Quercetin*) received 25 mg/kg/day tramadol IP and 100 mg/kg/day quercetin IP followed by 75 mg/kg tramadol IP on the 14th day. Group 8 (*Quercetin-Naloxone*) received 25 mg/kg/day tramadol IP for 13 days and 75 mg/kg tramadol IP on the 14th day followed by 100 mg/kg quercetin IP, 15 min after tramadol injection and 2 mg/kg naloxone IV followed by 4 mg/kg naloxone injection once every hour for 6 h (Fig. 1). Rats were kept in constant room temperature [22 ± 2 °C, light/dark 12 h intervals] during the 6 h evaluation period and after that. In addition, they were under the direct observation of the researcher during the 6 h.

The rats were clinically monitored for 6 h on the final experimental day, and the number, duration, and severity of seizures and sedation level were recorded. The sedation level was assessed based on a 4-point scale, ranging from 0 to 3. In stage 0 (awake), rats were fully awake and active (engaged in locomotion, rearing, head movements, or grooming), and their gait and reflexes were entirely intact. In stage 1 (lethargic), rat activities were reduced (little locomotion, rearing, or grooming), and they experienced mild gait abnormalities and reduced muscle tone, while the righting reflexes were normal. In stage 2 (mild comatose), rats were dormant and immobile, and the righting reflexes decreased. In stage 3 (deep coma), rats were comatose and had no righting reflexes [4]. The righting reflex was measured by placing the animal on its back to see its ability to stand or return to normal [29].

Seizure severity was rated according to the criteria of Racine. In stage 1, rats were immobile, with closed eyes and facial clonus, while the hair around the nose was shaking. In stage 2, rats experienced head-shaking accompanied by more severe facial clonus. In stage 3, rats exhibited forelimb clonus. In stage 4, rats rose on their hind legs and exhibited bilateral forelimb clonus. In stage 5, rats rose on their hind legs, had no balance, fell, and underwent generalized clonic seizures [4]. Low-level seizures often only included a single stage, but some high-level seizures were advanced between stages, and they were scored as the highest level reached. However, seizures occurred in a short time, thus quickly reaching their highest level. After drug injections on day 14, the rats were placed individually in glass boxes to assess their movement and activities. After drug administration on day 14, each animal was placed in identical wooden boxes. Three walls and the box floor were painted white, and the other side of the plate glass allowed animals to be observed. Each animal received a unique identification number that did not identify individual treatment conditions. A video camera recorded all behavioral tests connected to a video recorder. A trained observer subsequently analyzed videos. At the end of the behavioral recording, rats were released and cared for at the BUMS animal house.

While the experimenter who conducted injections was aware of the identity of the study groups, the observer who carried out statistical analysis and outcome assessment (scored the seizures and sedation level) was blinded to the group allocation by assigning different numbers to each group. Collected data were analyzed using SPSS software 16. The normality of quantitative variables was determined using Shapiro–Wilk tests. One-way ANOVA, Bonferroni post hoc, Kruskal–Wallis, Dunn-Bonferroni post hoc, and regression analysis were used to compare quantitative parameters and chi-square tests to compare qualitative parameters. The average seizure score and the mean number of seizures during the 6-h observation period in each group were compared. The generalized estimating equation (GEE) model was employed to analyze the longitudinal variables measured at different time points. In the GEE model, longitudinal variables were entered into the model as dependent variables and groups as explanatory variables. As a dependent variable, the sedation level was measured and recorded immediately, at 15 min (before any other drug injection), 30 min, 1 h (h), 2 h, 3 h, 4 h, 5 h, and 6 h after tramadol injection. A p-value less than 0.05 was considered significant.

## Results

One death occurred in each of the chronic Quercetin, Quercetin-Naloxone, and Diazepam-Naloxone groups during the study. The deaths occurred during day 14 (after the administration of corresponding treatments). At baseline, rat weights did not differ between groups (F = 0.4, df = 7, p = 0.88). However, at the end of the study, the weight of the chronic quercetin group was significantly lower than that of the tramadol and control groups (F = 2.2, df = 7, p = 0.04) (Table 1).

**Table 1:** Weight of rats in different groups before and after the experiments

Groups	Control	Tramadol	Tramadol + Naloxone	Tramadol + Diazepam
Rat weight before interventions (g)	219.1 ± 9.02	216.8 ± 15.5	211.6 ± 25.9	222.0 ± 19.3
Rat weight after interventions (g)	271.7 ± 15.7	271.6 ± 20.8	253.7 ± 26.0	275.5 ± 15.5

Groups	Tramadol + Naloxone + Diazepam	Tramadol + acute quercetin	Tramadol + chronic quercetin	Tramadol + acute quercetin + Naloxone
Rat weight before interventions (g)	209.0 ± 20.2	217.2 ± 21.5	216.7 ± 27.8	220.6 ± 18.2
Rat weight after interventions (g)	255.1 ± 27.5	265.2 ± 23.2	229.3 ± 33.7*,**	264.4 ± 52.4

^*^ *p* < 0.05 significant as compared to the control group

^**^ *p* < 0.05 significant as compared to tramadol group

### Number of seizure

**Fig. 2:**
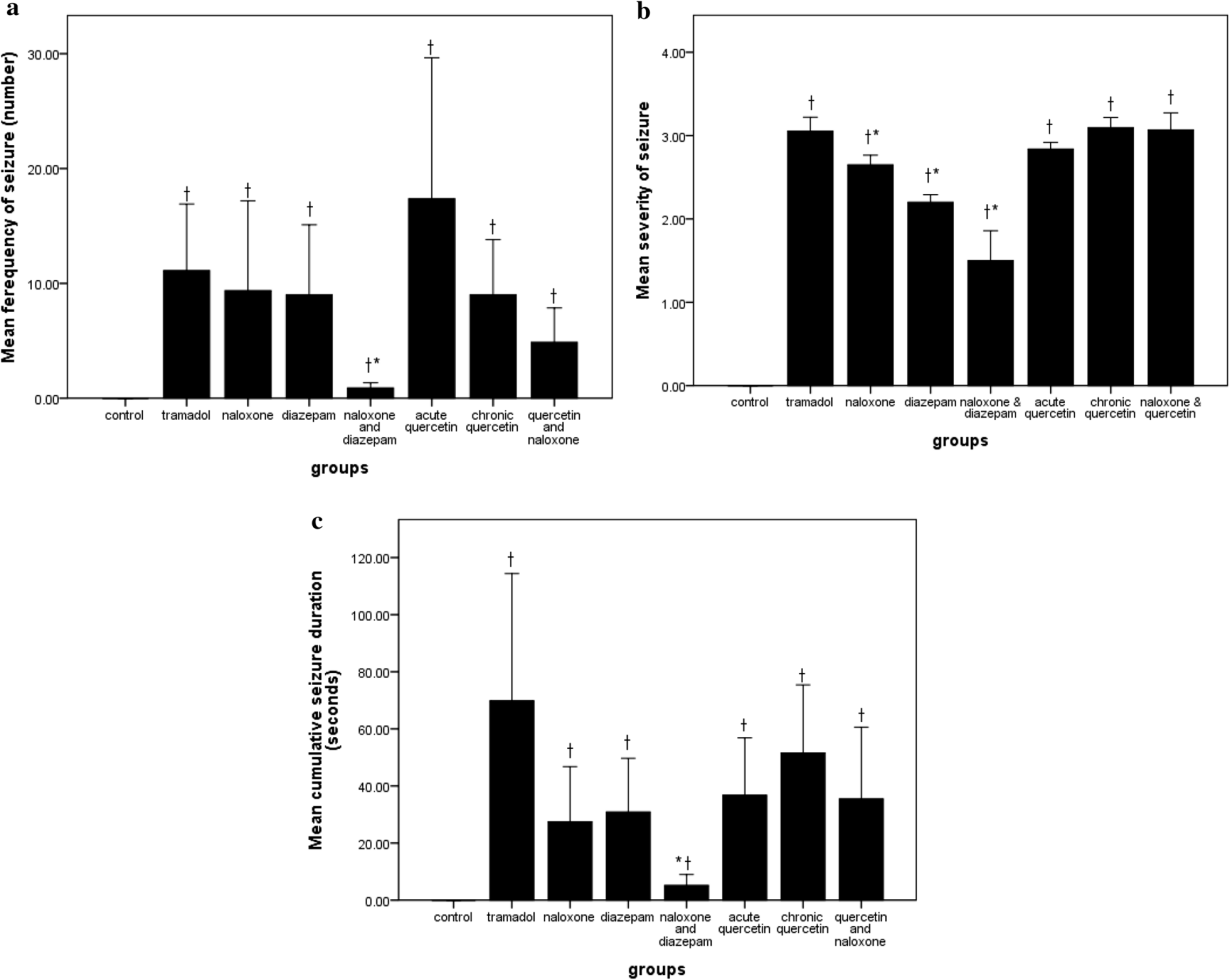
Comparison of the number (2a), severity (2b), and cumulative duration of seizures (2c) in the study groups. ^†^p < 0.05 compared to the Control group; ^*^p < 0.05 compared to the tramadol group, the values are Mean ± SE

The number of seizures for each animal was counted, and then the average of all seizures occurring in each group was calculated and compared (χ^2^ = 15.2, df = 7, p = 0.04). No animal in the control group seized. As seen in Fig. 2a, the number of seizures in all experimental groups was significantly higher than that of the control group. However, the rats in the naloxone-diazepam group had a significantly decreased incidence of seizures compared to rats in the tramadol group (P < 0.05) (Fig. 2a).

### Severity of seizure

Animals in each different group had different numbers of seizures with various severities and duration. The averages of all seizure scores observed in each group were compared (F = 15.46, df = 7, P < 0.001) (Fig. 2b). The Naloxone, Diazepam, and Naloxone-Diazepam groups showed significantly reduced seizure severity, compared to the Tramadol group.

### Cumulative seizure duration

The cumulative time of seizing during 6 h was calculated in each rat and the mean duration time for the rats in each group was calculated and compared between the groups (χ^2^ = 14.7,df = 7, p = 0.04). All groups experienced significantly higher cumulative seizure duration, compared to the control group (p < 0.05) naloxone-diazepam group showed significantly reduced cumulative time of seizure that each animal involved, compared to the tramadol group (Fig. 2c).

**Fig. 3:**
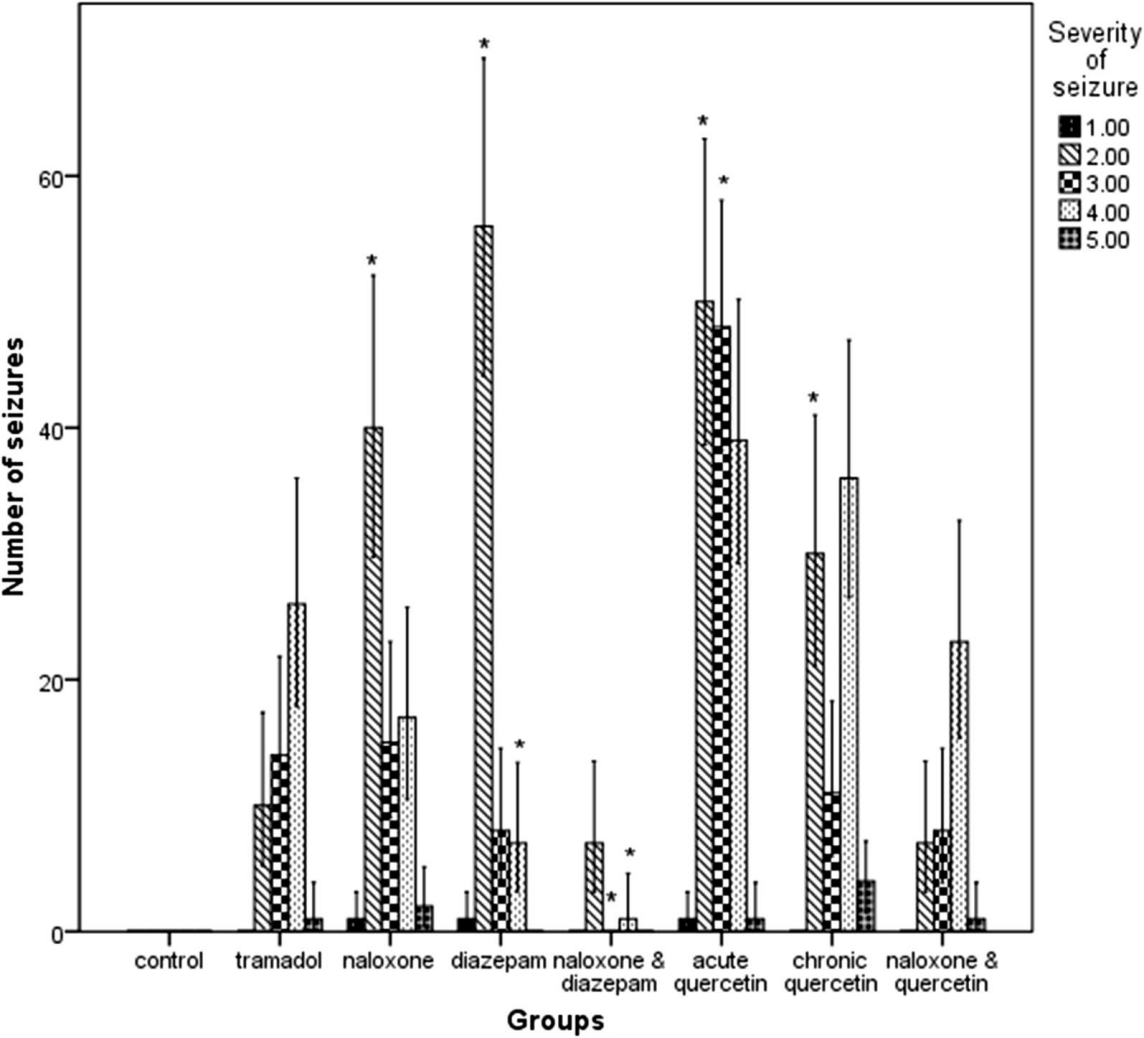
Comparison of the number of seizures according to their severity in the study groups. *p < 0.05 compared with the Tramadol group, the values are Mean ± SE

**Fig. 4:**
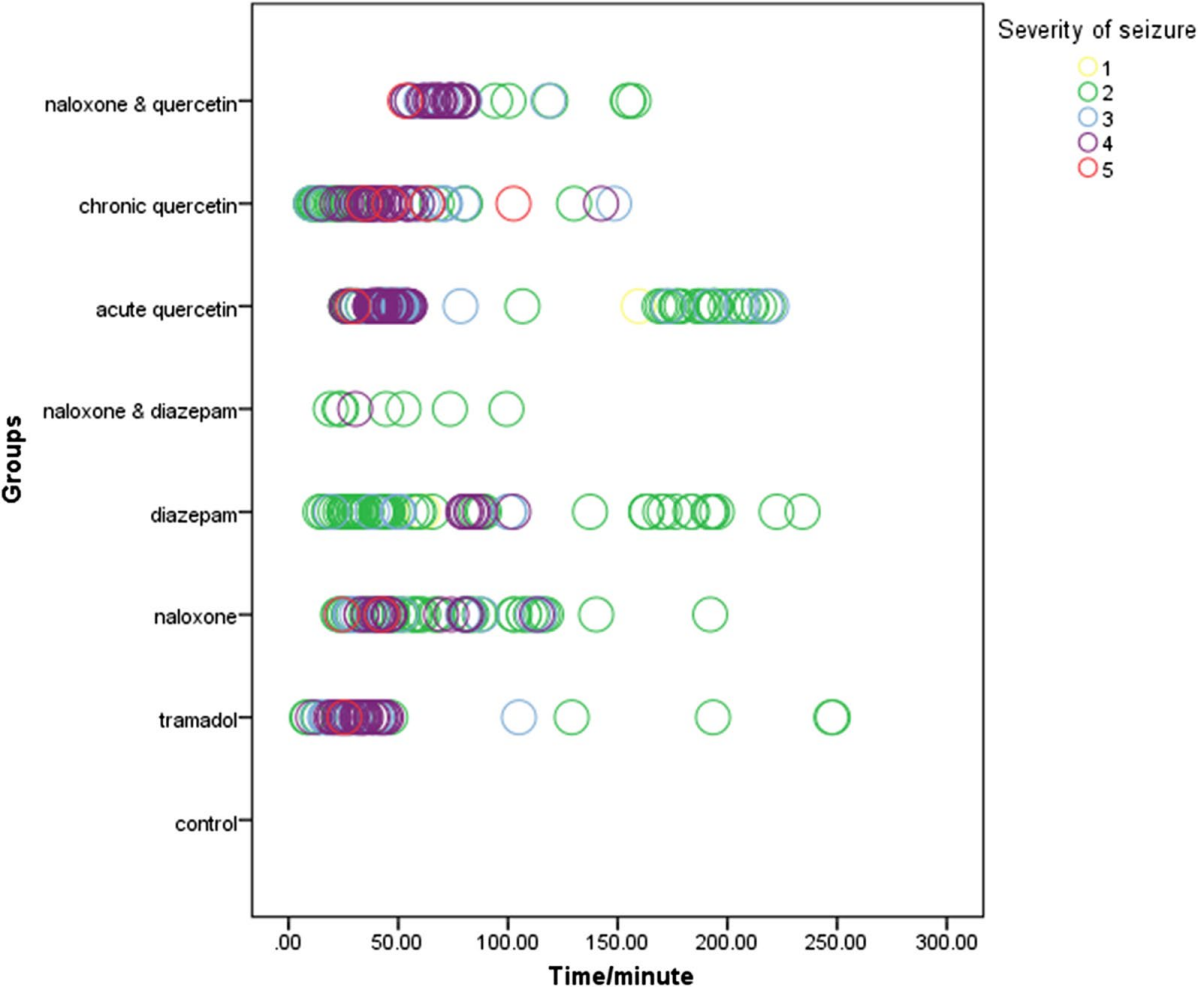
Time-course of seizures clustered by severity in the different groups

**Fig. 5:**
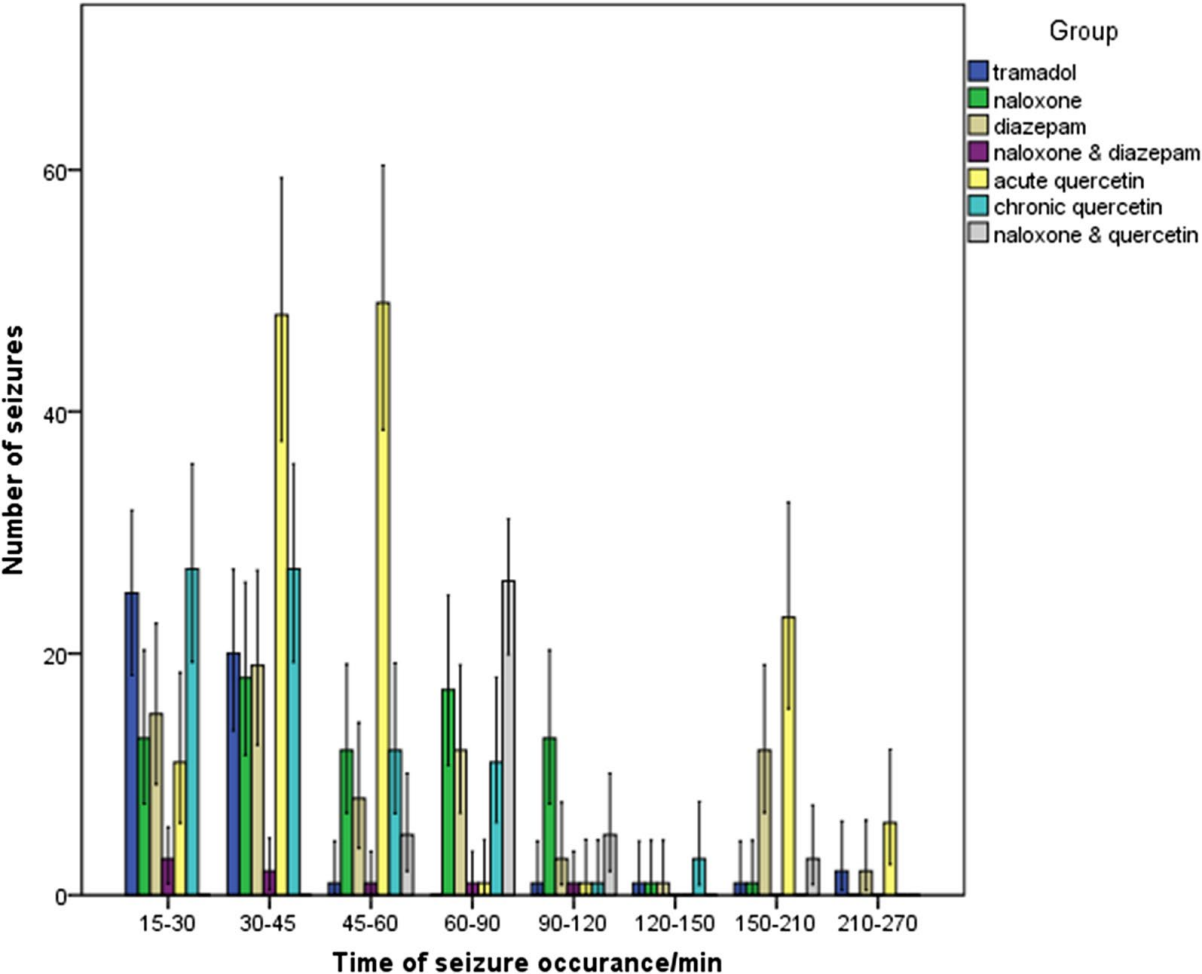
The number of seizures occurring during 6 h in the different groups

The number of moderate (level 3) (χ^2^ = 14, p < 0.001) and severe (level 4) (χ^2^ = 23.15, p < 0.001) seizures in the naloxone-diazepam group was significantly lower than that of tramadol group Diazepam was associated with a significantly higher ratio of mild seizures (level 2) (χ^2^ = 32.06, p < 0.001) and a lower proportion of severe seizures (level 4) (χ^2^ = 10.94, p < 0.001) compared to tramadol group. The acute Quercetin group showed a significantly higher number of low-intensity (level 2) (χ^2^ = 26.67, p < 0.001) and moderate (level 3) (χ^2^ = 18.64, p < 0.001) seizures than the tramadol group. Chronic quercetin group (χ^2^ = 10.00, p = 0.001) and naloxone group (χ^2^ = 10.2, p = 0.001), compared to tramadol, only significantly increased the number of low-intensity (level 2) seizures (Fig. 3). The Time course of seizure occurrence in the different groups is shown in Figs. 4 and 5. The latency to first seizure was not significantly different between groups (F = 0.6, df = 7, p = 0.72).

Of note, each animal in each different group exhibited different numbers of seizures with various severities and durations. The duration and severity of seizures were included in the regression analysis. The results of regression analysis showed that seizure severity in the naloxone (P = 0.03, β = − 0.40), diazepam (P < 0.001, β = − 0.85), and naloxone-diazepam (P < 0.001, β = 11.55) groups decreased significantly, in comparison with the tramadol group. The duration of seizures in the naloxone (P < 0.001, β = − 4.22), diazepam (P < 0.001, β = − 3.78), and acute quercetin (P < 0.001, β = − 5.20) groups decreased significantly compared to the tramadol group (Table 2).

**Table 2:** Results of regression analysis (seizure severity and seizure duration) in the different groups

Variable		B	SE	t	p-value
Severity of seizures	Naloxone vs tramadol	− 0.40	0.18	2.19	0.03
	Diazepam vs tramadol	− 0.85	0.18	2.19	< 0.001
	Naloxone-diazepam vs tramadol	− 1.55	0.33	− 4.65	< 0.001
	Acute quercetin vs tramadol	− 0.21	0.16	1.29	0.20
	Chronic quercetin vs tramadol	0.04	0.18	0.23	0.81
	Quercetin-naloxone vs tramadol	0.01	0.21	0.07	0.94
	Tramadol vs control	3.05	0.37	8.16	< 0.001
	Naloxone vs control	2.65	0.37	7.22	< 0.001
	Diazepam vs control	2.20	0.36	5.98	< 0.001
	Naloxone-diazepam vs control	1.50	0.46	3.27	0.001
	Acute quercetin vs control	2.84	0.35	7.93	< 0.001
	Chronic quercetin vs control	3.07	0.36	8.47	< 0.001
	Quercetin-naloxone vs control	3.09	0.38	8.05	< 0.001
Duration of seizures	Naloxone vs tramadol	− 4.22	1.13	− 3.73	< 0.001
	Diazepam vs tramadol	− 3.78	1.14	− 3.32	< 0.001
	Naloxone-diazepam vs tramadol	− 3.15	2.05	− 1.54	0.12
	Acute quercetin vs tramadol	− 5.20	1.02	− 5.11	< 0.001
	Chronic quercetin vs tramadol	− 1.69	1.11	− 1.52	0.13
	Quercetin-naloxone vs tramadol	− 0.61	1.29	− 0.48	0.63
	Tramadol vs control	7.07	2.29	3.09	0.002
	Naloxone vs control	2.84	2.24	1.27	0.20
	Diazepam vs control	3.29	2.25	1.46	0.14
	Naloxone-diazepam vs control	3.92	2.81	1.39	0.16
	Acute quercetin vs control	1.87	2.19	0.85	0.39
	Chronic quercetin vs control	5.37	2.24	2.40	0.02
	Quercetin-naloxone vs control	6.45	2.33	2.77	0.005

### Sedation level

**Fig. 6:**
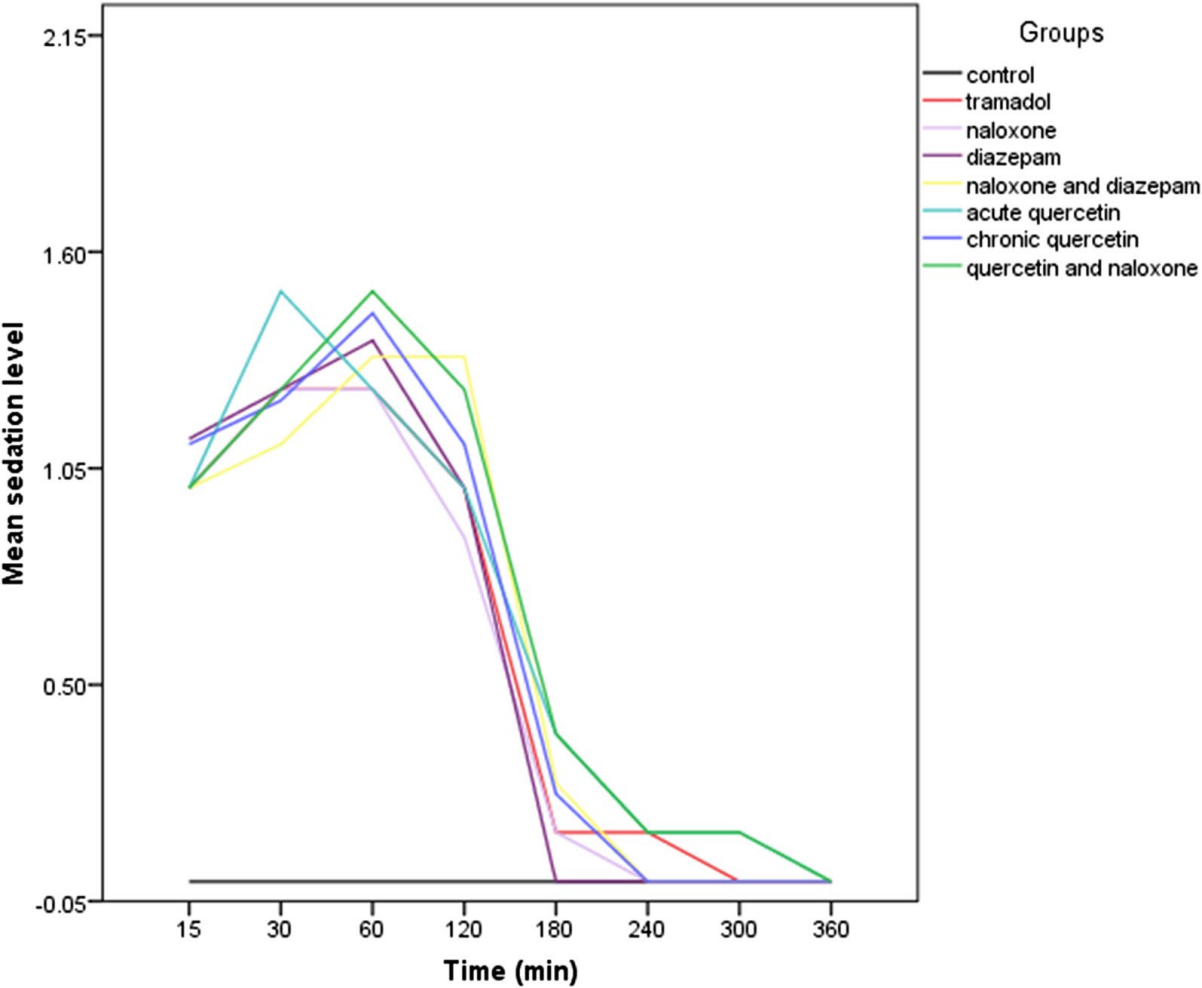
Time-course of sedation levels in the different treatment groups

In the GEE model, the *sedation level* was considered as a dependent variable and *the group* as an explanatory variable. All groups had a higher sedation level than the Control group. The tramadol group showed a 0.59 higher sedation level in the GEE model analysis than the Control group (P < 0.05). The quercetin-naloxone group showed 0.70 more sedation than the control group (P < 0.001); this group had the highest increase in sedation level. However, various treatments' sedation effects were not significantly different from the tramadol group (Table 3). The mean sedation level was significantly different between groups in 15 min to 2 h after tramadol administration, in all cases being greater than control (Table 4). The highest sedation level occurred at 30 to 60 min and approached zero starting at 3 h after tramadol administration (Fig. 6).

**Table 3:** Comparison of sedation levels in the different groups vs the Control and Tramadol groups based on the GEE model

Groups	B	SE	Wald	p-value
Tramadol vs control	0.59	0.04	192.53	< 0.001
Naloxone vs control	0.56	0.04	162.00	< 0.001
Diazepam vs control	0.59	0.04	192.53	< 0.001
Naloxone-diazepam vs control	0.64	0.06	100.04	< 0.001
Acute quercetin vs control	0.67	0.07	93.03	< 0.001
Chronic quercetin vs control	0.64	0.04	194.32	< 0.001
Quercetin-naloxone vs control	0.70	0.07	113.28	< 0.001
Naloxone vs tramadol	− 0.03	0.06	0.25	0.61
Diazepam vs tramadol	− 5.06 e−18	0.06	0.0001	1.00
Naloxone-diazepam vs tramadol	0.04	0.08	0.32	0.57
Acute quercetin vs tramadol	0.08	0.08	0.91	0.34
Chronic quercetin vs tramadol	0.04	0.06	0.52	0.47
Quercetin-naloxone vs tramadol	0.11	0.07	1.93	0.16

**Table 4:** Comparison of mean sedation levels in the different treatment groups during the 6-h course following tramadol administration

Groups/times	15 min	30 min	1 h	2 h	3 h	4 h	5 h	6 h
Control	0 ± 0	0 ± 0	0 ± 0	0 ± 0	0 ± 0	0 ± 0	0 ± 0	0 ± 0
Tramadol	1.0 ± 0.0	1.3 ± 0.4	1.3 ± 0.4	1.0 ± 0.0	0.1 ± 0.3	0.1 ± 0.3	0 ± 0	0 ± 0
Naloxone	1.0 ± 0.0	1.3 ± 0.4	1.3 ± 0.4	0.8 ± 0.3	0.1 ± 0.3	0 ± 0	0 ± 0	0 ± 0
Diazepam	1.1 ± 0.4	1.3 ± 0.4	1.4 ± 0.5	1 ± 0	0 ± 0	0 ± 0	0 ± 0	0 ± 0
Naloxone-diazepam	1.0 ± 0.0	1.1 ± 0.3	1.3 ± 0.5	1.3 ± 0.5	0.3 ± 0.4	0 ± 0	0 ± 0	0 ± 0
Acute quercetin	1.0 ± 0.0	1.5 ± 0.5	1.3 ± 0.4	1.0 ± 0.5	0.4 ± 0.5	0.1 ± 0.3	0.1 ± 0.3	0 ± 0
Chronic quercetin	1.1 ± 0.3	1.2 ± 0.4	1.4 ± 0.5	1.1 ± 0.3	0.2 ± 0.4	0 ± 0	0 ± 0	0 ± 0
Quercetin-naloxone	1.0 ± 0.0	1.2 ± 0.4	1.5 ± 0.5	1.3 ± 0.4	0.4 ± 0.5	0.1 ± 0.3	0.1 ± 0.3	0 ± 0
Test results	F = 35.6 P = 0.0	F = 8.1 P = 0.0	F = 8.9 P = 0.0	F = 7.8 P = 0.0	F = 1 P = 0.4	F = 0.7 P = 0.6	F = 0.8 P = 0.5	

The values are mean ± standard deviation

## Discussion

Our study demonstrated that the naloxone-diazepam combination reduces the number, severity, and duration of tramadol-induced seizures in acute-on-chronically tramadol-treated rats. However, it did not improve sedation.

Naloxone, the antidote used to reverse opioids overdose in humans, is a competitive opioid receptor antagonist. It has been shown to potentiate the anticonvulsant effects of benzodiazepines and barbiturates, thus exerting mild anticonvulsant effects [30]. Lagard et al. (2017) evaluated the effectiveness in the rat of naloxone and diazepam on tramadol-induced seizures and respiratory depression using EEG and plethysmography, respectively, after the acute single-administration of 75 mg/kg of tramadol. They showed that the diazepam-naloxone combination is the most effective treatment for relieving tramadol-induced seizures and improving ventilation. In the present study, with acute-on-chronic tramadol exposure, naloxone reduced the number and duration of seizures.

Nevertheless, contradictory results have been reported regarding naloxone-attributed effects on tramadol poisoning and seizures. Similar to the present study, some studies have shown protective effects of naloxone against opioid- and non-opioid-induced seizures. Naloxone administration reduced tramadol-induced seizure activity in mice treated with pentylenetetrazole [31]. These authors proposed an opioid-dependent GABAergic inhibitory pathway to explain tramadol-induced seizures. Similarly, another study showed that naloxone significantly reduced seizures induced by meperidine and its seizurogenic metabolite normeperidine in mice [32]. Moreover, 12 h of continuous naloxone infusion in a rat model of kainic acid-induced seizures resulted in dose-dependent protective effects [33].

In humans, seizures have been attributed to naloxone as an adverse effect in opioid-poisoned patients [16]. This has been attributed to the potential disruptive effects of other associated toxins and to withdrawal in opioid-dependent individuals; it has also been seen as a consequence of long-term hypoxia [4, 34]. Lagard et al. showed that naloxone increased the number of seizures and prolonged their onset but reduced respiratory depression [4]. In another study in mice, tramadol-induced seizures occurred mainly with high doses of naloxone [35]. Experimental data on seizures resulting from naloxone administration may vary depending on the animal model and experimental conditions. According to a meta-analysis of human studies, naloxone administration was not associated with seizures [36].

In the present study, diazepam reduced the severity and duration of seizures in acute-on-chronically exposed rats. While it increased the number of mild seizures, yet significantly reduced the number of severe seizures. In line with the present study results, Lagard et al. (2017) also showed that diazepam relieved tramadol-induced seizures but significantly deepened the sedation level in rats [4]. The same group examined the effects of 20 mg/kg subcutaneous diazepam administration on tramadol neurotoxicity in rats showing that diazepam did not increase tramadol-induced mortality but significantly altered the pattern of toxicity by preventing seizures and enhancing respiratory depression [3]. Some studies have reported that patients who abuse tramadol along with benzodiazepines have a lower rate of seizures, though not statistically significant, than individuals who abuse tramadol along with heroin [37]. Some reports did not consider anticonvulsant prophylaxis to be necessary in patients overdosed with tramadol because the treatment of tramadol-induced seizures with benzodiazepines may have additive or synergistic effects on the GABA receptor leading to worsened depression of consciousness [17]. Several reports suggested that benzodiazepines, even at therapeutic doses, may increase the morbidity and mortality of tramadol overdose [19, 38], possibly due to the cumulative CNS depressive effects.

In our study, quercetin administration had no significant protective effects against seizures. Acute quercetin use, increased the number of mild-to-moderate tramadol-induced seizures. Chronic quercetin administration significantly increased the number of mild episodes. As far as we know, quercetin effectiveness in tramadol-induced seizures has not been studied until now. However, its protective effects against other drug-induced seizures have been reported. For example, quercetin reduced kainic acid-induced seizure score in a dose-dependent manner [20]. The anticonvulsant potential of quercetin against 6 Hz-induced convulsive seizures has been demonstrated at doses of 10 to 200 mg/kg, the highest quercetin-related anticonvulsant activity being associated with high plasma and cerebral concentrations [24]. The exact anticonvulsant effects of quercetin and other flavonoids is not known, possibly mediated by their antioxidant properties [39, 40], interaction with GABA receptors, glycine [41], acetylcholine [24], serotonin receptors, [42] or adenosine [43]. Understanding the exact clinical effects of quercetin in tramadol overdose warranted further studies. Based on previous literature, quercetin is not carcinogenic. Quercetin supplements are commercially available in some countries [44, 45] and the effects of quercetin supplements have been examined in clinical trials [46, 47]. Their safety for human use is generally accepted [44, 45].

Our empirical study has limitations, including the fact that the generalization of animal findings to humans should always be done cautiously. These findings can only be considered as a model of the complex clinical condition in humans. However, our model exhibited tramadol-induced decreased level of consciousness and seizures similar to that reported in humans [48–50]. We realize that therapeutic studies in animals can generate hypotheses for what may happen in human clinical use. Assessing the validity of any of such hypotheses requires human data. In this study, we utilized single-dose treatments. Further research may be of utility by generating dose–response data. Further experimental and clinical research is needed to demonstrate the molecular mechanism of the effect of these treatments on tramadol overdose.

## Conclusion

The combination of naloxone and diazepam in acute-on-chronic tramadol poisoning can effectively reduce the number, severity, and duration of seizures in the rat. This combination also decreases the number of severe seizures compared to other treatments, including naloxone alone, diazepam alone, and quercetin. However, none of the tested treatments improves sedation. This is important because patients who are using tramadol chronically may intentionally or accidentally overdose. Our data utilized an acute on chronic model and may be less applicable to patients who overdose on tramadol without prior use.

## Data Availability

The datasets are available from the corresponding author on formal and logical request.

## Abbreviations

GEE: Generalized estimating equation
EEG: Electroencephalography
GABAA: γ-Aminobutyric acid type A
NMDA: *N*-methyl-d-aspartate
DMSO: Dimethyl sulfoxide
IP: Intraperitoneal
CNS: Central nervous system
